# Family satisfaction and self-efficacy among low-income adolescents during the COVID-19 pandemic: A comparative analysis of parents' educational attainment

**DOI:** 10.3389/fpsyt.2022.942927

**Published:** 2022-07-26

**Authors:** Jaewon Lee, Jennifer Allen, Hyejung Lim, Gyuhyun Choi, Jiyu Jung

**Affiliations:** ^1^School of Social Work, Inha University, Incheon, South Korea; ^2^School of Social Work, Michigan State University, East Lansing, MI, United States; ^3^School of Education, Korea University, Seoul, South Korea; ^4^Integrative Arts Therapy, Dongduk Women's University, Seoul, South Korea; ^5^Korea Development Bank Foundation, Seoul, South Korea

**Keywords:** low-income adolescents, family satisfaction, self-efficacy, parents' educational attainment, COVID-19

## Abstract

**Purposes:**

Given that the period from middle to high school is important to develop and cultivate self-efficacy, reduced support in low-income families might negatively influence the development of self-efficacy among low-income students since COVID-19. This study aims to investigate the association between family satisfaction and self-efficacy among low-income students since COVID-19 and the moderating effect of parents' educational attainment on the relationship.

**Methods:**

255 low-income students in South-Korea were selected for the final sample. The PROCESS macro 3.4 for Statistical Product and Service Solutions was used to analyze the data.

**Results:**

Family satisfaction was positively related to self-efficacy among low-income students. There was a significant moderating effect of parents' educational attainment on the relationship between family satisfaction and self-efficacy among low-income students during the COVID-19 pandemic.

**Discussion:**

Financial support and COVID-19 benefits should be prioritized to low-income families with adolescents to improve family relationships, leading to increase self-efficacy among low-income students. Social welfare programs targeting family relationships in low-income households should be especially targeted toward low-income households without a parent who received higher education. Life-long education should be provided to parents in low-income families who did not gain higher education as their educational attainment influences the self-efficacy of their adolescent children.

## Introduction

From January 2020 to June 2022, there have been more than 18 million confirmed cases of COVID-19 in South Korea, with more than 24,000 associated deaths (1). COVID-19-related school closures for 5 weeks from March to April 2020 drastically disrupted the lives of South Korean adolescents (2). From early April to late May or early June, depending on the students' grade, they proceeded to have fully online schooling (2). During this time, researchers found that high self-efficacy was important for adolescents' positive adaptation and functioning (3, 4).

### Self-efficacy in adolescence

Self-efficacy is a person's perceived capability to achieve something or complete a task (5, 6). Adolescence is an important developmental period for self-efficacy, affected by parental, school, and peer influences (7). Self-efficacy is also inversely associated with income and savings (5, 6, 8–10). A few studies have examined self-efficacy among low-income adolescents, and adolescents in households with higher incomes tend to report higher self-efficacy of many types than those in lower-income households (5, 6, 10). Among Chinese adolescents aged 12–18 years, family's combined annual income was positively correlated with adolescents' general self-efficacy (5). In another sample of Chinese high schoolers, students who were poor (i.e., a monthly income of less than $56 per person) had significantly lower general self-efficacy than those who were not (6). Thus, there is some evidence to suggest that self-efficacy differs by family income among adolescents, but more research is needed.

Some research has investigated the importance of self-efficacy in adolescence and beyond during the COVID-19 pandemic. In a sample of Italian adolescents surveyed during the nationwide COVID-19-related lockdown, researchers found that both emotional and self-regulated learning self-efficacy positively predicted adolescents' subjective wellbeing and positive coping during the lockdown (3). Moreover, in a sample of parent-child dyads that included elementary-aged, pre-adolescent and adolescent children, youth who had greater general self-efficacy had a smaller increase in mental health symptoms during the COVID-19 outbreak, as compared to data collected before (4). In data collected from Chinese high school students in April 2020, two types of self-efficacy (internet self-efficacy and self-efficacy of interacting with learning content) were positively associated with positive mind states related to online learning as well as students' perceived effectiveness of online learning (11).

Two studies examined the importance of self-efficacy among university students during COVID-19. In March 2020, a measure of academic self-efficacy was inversely associated with anxiety among Spanish university students (12). In spring semester 2021, academic self-efficacy significantly predicted Korean university student's engagement in their hybrid learning (13). Last, a few studies examined the importance of self-efficacy during COVID-19 among adults of all ages. In a longitudinal study of French adults conducted over eight weeks of COVID-19-related lockdown, higher self-efficacy was associated with higher positive affect (14). Similarly, in a sample of Italian adults, emotion regulation self-efficacy was inversely associated with adults' anxiety and depressive symptoms during COVID-19, and in a sample of Turkish adults, self-efficacy related to COVID-19 prevention was associated with better mental health outcomes (15, 16). Thus, research has examined the importance of self-efficacy for academic and mental health outcomes during COVID-19, but more research is needed on adolescents, as much research has looked at adults.

### Adolescent self-efficacy and parental educational attainment

Compared to adolescents whose parents have higher educational attainment, adolescents with parents who have low or no higher educational attainment also have lower perceived self-efficacy in most studies (5, 11, 12). For example, in a sample of Chinese adolescents aged 12 to 18 years, both mothers' and fathers' educational attainment were positively correlated with adolescents' general self-efficacy scale score (5). Additionally, in a large, nationally representative sample of American 10th graders, family socioeconomic status [SES] – mothers', fathers', and/or guardians' educational attainment - was positively associated with math and English self-efficacy (11). Moreover, in a longitudinal study of Polish adolescents, when mothers' educational level increased over time, so did their adolescent children's general self-efficacy scores (12). In another sample of regular and vocational high school students in China, students in regular high schools reported significantly higher maternal and paternal educational attainment (13). Although parental educational attainment and adolescent self-efficacy were not directly compared, students in regular high schools, whose parents had higher educational attainment, reported higher self-efficacy than the vocational high school students (13). In another sample of African American high school students, those who reported higher parental SES – including parental educational attainment – reported higher career self-efficacy than adolescents with lower parental SES (14). Moreover, among American 9th graders, students whose mothers did not obtain higher education had lower coping self-efficacy, or belief in their own ability to deal with stress (15). Despite the positive correlations found between parental educational attainment and their adolescent children's self-efficacy, other co-variates such as cultural capital should be considered to explain the relationship between the two variables (7).

Only two studies were located that examined the relationship between self-efficacy and parental educational attainment during adolescence and young adulthood in the context of the COVID-19 pandemic; none were located looking at adolescents only (17, 18). First, general self-efficacy was not significantly associated with parental education in a sample of 16–25-year-olds in the U.K. in a survey collected from February to October 2021 (18). Second, researchers surveyed university students in December 2020, after the students returned to campus, and the authors found that parental education predicted students' perceived self-efficacy to prevent COVID-19 infection (17).

### Self-efficacy and family satisfaction

A few studies have examined the relationship between self-efficacy and family satisfaction in adolescent and adult samples (16, 19, 20). In a sample of young to elderly adults, family satisfaction was associated with self-efficacy to manage work-family conflict (20). Moreover, two studies included the relationship between adolescents' family satisfaction and filial self-efficacy, the latter of which was defined as “perceived capability to exercise their expanding agentic role in their relationships with their parents” (19). In the first study, Italian adolescents with higher perceived filial self-efficacy reported higher family satisfaction at baseline and 2 years later (19). In the second study, researchers used data from parent-adolescent dyads to determine that adolescents' filial self-efficacy was associated with family satisfaction through collective family efficacy, which included the filial self-efficacy variable as well as dyadic parent-child efficacy and dyadic spousal efficacy (16). As only a few studies were located examining this relationship, more research is needed on the relationship between adolescents' family satisfaction and self-efficacy.

Self-efficacy and factors related to family satisfaction were also examined in the context of the COVID-19 pandemic. In a sample of mothers whose children (kindergarten through grade 2) engaged in schooling from home during lockdowns, mothers' self-efficacy related to teaching was inversely associated with mother-child conflict during schooling, as well as positively associated with mothers' perceived mother-child closeness (21). Further, parenting self-efficacy was inversely associated with family functioning in a sample of families with children in first grade during the COVID-19-related lockdowns in Italy (22). Thus, more research is needed examining family satisfaction and other dynamics in families with adolescent children in the context of COVID-19.

### Family satisfaction and parental educational attainment

Two studies were found that examined variables related to family satisfaction and parental educational attainment (23, 24). First, in the only study found to directly examine the relationship between adolescents' self-reported family satisfaction and parental educational attainment, adolescents living in remarried families reported higher satisfaction with their parent and stepparent if either their parent or stepparent had higher educational attainment (23). Additionally, family members' higher educational attainment was positively associated with more frequent family communication, two means of which (face-to-face and phone) were positively associated with perceived family wellbeing (24). The family wellbeing variable included aspects of family harmony and happiness, which may be associated with family satisfaction (17, 25).

Two studies were located that examined family dynamics and parental educational attainment among families with adolescent children during COVID-19. In a sample of parents of children aged 3–17 years in March 2020 in Wuhan and Shanghai, China, parents with a higher educational attainment (bachelor's degree or above) were less irritable toward their children and reported higher closeness with their children than parents with lower educational attainment (high school or below) (26). Second, during the COVID-19 pandemic, adolescents in the Midwestern US who had parents with low or moderate levels of education (a bachelor's degree or below) had a more significant increase in family stressors during the pandemic and associated lockdowns than did adolescents with parents with a high level of education (a graduate degree) (27). Thus, more research is needed on this relationship with families with adolescent children in the context of COVID-19.

### The present study

Evidence suggests that self-efficacy differs by household income or SES (5, 6). Since coronavirus disease has spread around the world, low-income families have experienced more economic difficulties, leading to lower support for their children. Given that the period from middle to high school is important to develop and cultivate self-efficacy, reduced support in low-income families might negatively influence the development of self-efficacy among low-income students since COVID-19. Similarly, research has shown that self-efficacy differs among adolescents based on their parents' educational attainment (5, 11, 12). Another variable affected by parental educational attainment is family satisfaction (23, 24), which thereby may be associated with self-efficacy (16, 19, 20). Individuals who are satisfied with their family members may be more likely to have higher self-efficacy based on emotional communication and support (16, 19, 20). Since the onset of the COVID-19 pandemic, family relationship quality and satisfaction may have been negatively affected by restrictions and stressors related to the pandemic (18, 28). Parents' educational attainment may also influence the relationship between family satisfaction and self-efficacy as higher education is helpful for communication skills, helping children with their own educational needs, and providing access to additional sources of knowledge and information (29, 30). Although some research has found relationships between these constructs among adolescents, we know of no study that examined the moderating impact of parental educational attainment on family satisfaction and self-efficacy among low-income adolescents, particularly in the context of the COVID-19 pandemic. Thus, this study aims to (1) investigate the association between family satisfaction and self-efficacy among low-income students since COVID-19; and (2) explore the moderating effect of parental educational attainment on the relationship. Based on these, we hypothesized that low-income adolescents who were satisfied with family relationships would have higher levels of self-efficacy and parental educational attainment would moderate the association between family satisfaction and self-efficacy among low-income adolescents.

## Methods

### Participants and sampling

Respondents in this study were middle and high school students in South Korea enrolled in a nationwide mentorship program, which was provided by the Korea Development Bank foundation, a non-profit organization. Students from low-income families were eligible to participate in the program, which was determined based on a poverty line. The poverty guideline was announced every year by the government, and the KDB foundation used it to recruit and choose low-income students. Data was collected in April 2021 through an online survey and students who did not respond initially were contacted again. Google Forms was used to implement the online survey and create a link to share with participants. To reach out to potential participants, we used contact information that was collected when students and their caregivers agreed to participate in the mentorship program. The questionnaire developed by the research team and refined by public school teachers and social workers was distributed to 264 low-income students *via* the link to access to the online survey. For distribution, we sent the online survey link to students via a text message, and all of the low-income students had a smartphone or cellphone on which to receive the message. Along with the survey, both low-income students and their caregivers received a consent form for participation. If either a participant or their caregiver declined to participate in the survey, the student was not included in this study. Participants who completed the survey obtained a $5 gift card for their participation. A total of 255 low-income students were selected for the final sample after some students or their caregivers declined to participate. Private information such as names or addresses was not collected in this study and the data does not include any identifiable information. Thus, this research was approved by the Institutional Review Board (#210216-2A).

### Measures

#### Self-efficacy

In this study, self-efficacy indicates a subjective belief regarding the ability to address difficulties someone may encounter. This was measured by the General Self-Efficacy Scale (GSE) (21). The GSE has been utilized in many countries, including South Korea, with good reliability and validity (22). This scale includes ten items: “I can always manage to solve difficult problems if I try hard enough”; “If someone opposes me, I can find the means and ways to get what I want.”; and “I can usually handle whatever comes my way.” Four response options (1 = Not at all true; 2 = Hardly true; 3 = Moderately true; 4 = Exactly true) were provided to respondents. The sum of the item was calculated and a higher score means higher self-efficacy. The Cronbach's alpha of the GSE in the current study was 0.90.

#### Family satisfaction

Participants were asked to report how much they were satisfied with their relationship with their family members. The family satisfaction scale developed by Olson was employed to measure it (31). The measure was a five-point Likert-type scale with 10 items. The five response options ranged from very dissatisfied to extremely satisfied. Question included: “The degree of closeness between family members”; “Your family's ability to cope with stress”; and “Family members concern for each other.” The 10 items were summed, and a higher sum indicated better family satisfaction. The measurement in the current study had a Cronbach's alpha of 0.94.

#### Parent's educational attainment

The educational attainment of respondents' parents was reported. Both their mothers and fathers reported their education level by selecting one of the following: “Middle school”; “High school”; “Bachelor's degree (Bachelor's, Associate's degree and some college)”; and “Graduate or professional degree.” If both mothers and fathers received further education than a high school diploma, they were considered to be in the group with higher education. On the other hand, the remaining group was regarded as those with non-higher education. For instance, a student whose father received a high school diploma and whose mother obtained a Bachelor's degree was regarded as being in the group with non-higher education.

#### Baseline variables

Middle and high schoolers' age, gender, academic performance, and whether they have siblings were included in this study. Given that respondents engaged in a mentorship program, the level of satisfaction with the program was controlled in the present study. The scale had seven items with a five-point Likert-type measurement and a Cronbach's alpha of 0.94.

#### Analysis strategies

**Figure 1:**
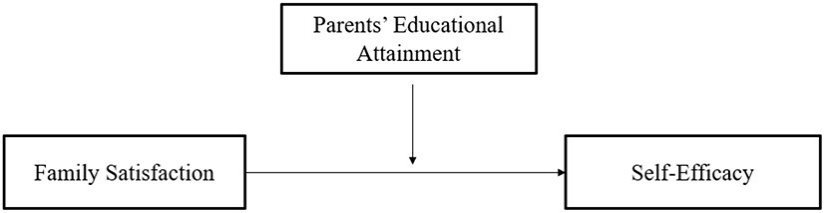
Research framework.

The PROCESS macro 3.4 for Statistical Product and Service Solutions (SPSS) was utilized to explore whether parents' educational attainment moderates the relationship between family satisfaction and self-efficacy among low-income students during COVID-19 pandemic. To test the moderating effect, a bootstrap approach using Model 1, suggested by Preacher and Hayes, was conducted at 95% bootstrap confidence intervals (32, 33). The research design used for this study is shown in Figure 1.

## Results

Descriptive statistics of the variables used in the current study are reported in Table 1. The summed scores for self-efficacy and family satisfaction were 29.83 and 38.85, respectively. Almost 40% of students had two parents who had both received higher education. Students' average age was 17.36, which may be adjusted to an international age of 16.36 as babies in Korea are considered 1 years old at birth. Approximately half of the total population were girls and the summed score of positive relationships with mentors was 31.03. About one-fourth of students had no siblings and student's average academic performance was C, with their average scores of primary classes being 7.66.

**Table 1:** Descriptive statistics.

**Variables**	**% or mean (SD)**
Self-efficacy	29.83 (5.58)
Family satisfaction	38.85 (8.43)
Parents' educational attainment	39.6%
Satisfaction with the program	31.03 (4.59)
Age	17.36 (1.75)
Academic performance	7.66 (3.73)
Gender (girl)	49.4%
Being an only child	24.3%

**Figure 2:**
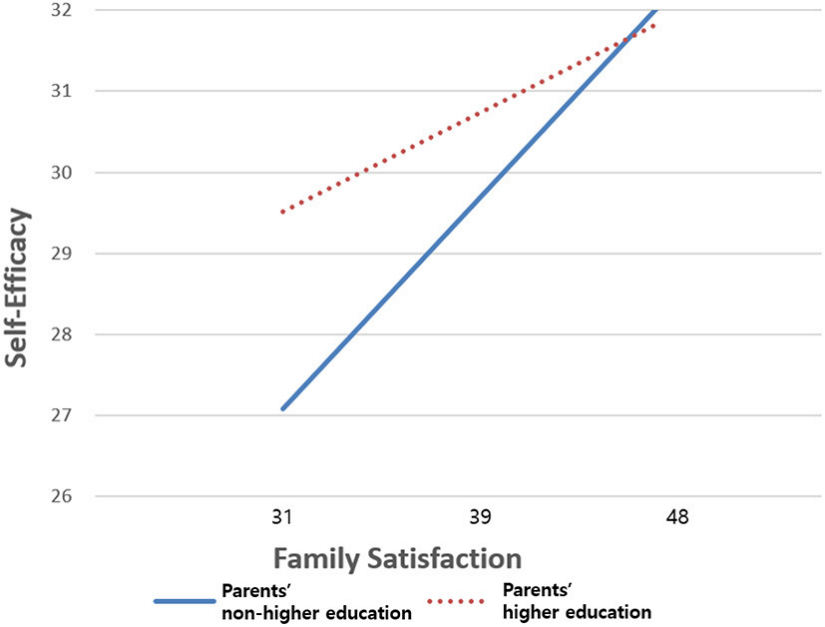
Moderating effect of parents' educational attainment on the relationship between family satisfaction and self-efficacy among low-income adolescents.

A moderating effect was found in Table 2. There was a significant moderating effect of parents' educational attainment on the relationship between family satisfaction and self-efficacy among low-income students during the COVID-19 pandemic (β = **-0.1**6, *p* **<** **0.0**5). In terms of a moderating effect, Figure 2 showed a specific difference between those whose parents received higher education and those whose parents did not. Regardless of parents' educational attainment, family satisfaction was positively associated with self-efficacy. That is, regardless of parental educational attainment, students showed higher self-efficacy if they have higher levels of family satisfaction. On the other hand, low-income students whose parents did not have higher education reported a lower self-efficacy score than those whose parents received higher education (27.08 vs. 29.52). The effect of parents' educational attainment was more strongly associated with self-efficacy among low-income students who had parents who did not receive higher education (4.96), while the effect was relatively minor for low-income students with parents with higher education (2.31). Moreover, parents' educational attainment and family satisfaction were positively related to self-efficacy among low-income students (β = 7.25, *p* < 0.05; β = 0.29, *p* < 0.001). Additionally, higher levels of students' academic performance and satisfaction with the program were related to higher self-efficacy (β = 0.29, *p* < 0.001; β = 0.16, *p* < 0.05).

**Table 2:** Moderating effects of parents' educational attainment on self-efficacy (unstandardized coefficients & standard error).

**Variables**	
(Constant)	11.19 (4.20)
Family satisfaction	0.29 (0.05)^***^
Age	0.01 (0.18)
Gender (girl)	−1.00 (0.63)
Academic performance	0.29 (0.08)^***^
Being an only child	0.36 (0.73)
Satisfaction with the program	0.16 (0.07)^*^
**Moderator**	
Parents' educational attainment	7.25 (3.03)^*^
**Moderating effect**	
Family satisfaction ^*^ Parents' educational attainment	−0.16 (0.08)^*^

* *p* < 0.05;

*** *p* < 0.001.

## Discussion

This study demonstrates how family satisfaction positively influenced self-efficacy among adolescents who have grown up in low-income households. During the COVID-19 pandemic, inequality in children's development has increased depending on parent's abilities. That is, low-income students with parents who have few resources might experience difficulties increasing their self-efficacy since COVID-19. This phenomenon existed before COVID-19 (1, 13, 14, 19), but it may have been even more evident since COVID-19 emerged. Thus, the current study included a comparative analysis to examine how parents' educational attainment influences the relationship between family satisfaction and self-efficacy among low-income adolescents since COVID-19. This research identified that parents' higher education moderated the association between adolescents' family satisfaction and self-efficacy. In other words, parents' educational attainment is important to increase self-efficacy among low-income students and family satisfaction is also key to increasing self-efficacy among low-income students whose parents did not receive higher education.

Since COVID-19 has spread around the world, vulnerable groups such as low-income families and adolescents have been more exposed to difficulties (26, 34). Given that low-income students might not have sufficient support for development of self-efficacy compared to those in middle or high economic classes, it is necessary to pay more attention to development of their self-efficacy. However, little evidence exists in understanding self-efficacy among low-income students. Generally, family satisfaction is related to self-efficacy (28–30), and this was confirmed in the current study, particularly among adolescents in low-income families. However, parents in low-income households might be busier and more exhausted in workplace since COVID-19 because an economic recession has made it more challenging to make money and be employed, particularly for low-income families (26). Unstable wages and job insecurity might interrupt communication or cohesion among or satisfaction with family members (27, 35). Therefore, low-income families might experience difficulties maintaining family relationships during COVID-19. This may negatively influence self-efficacy among low-income students as they feel they are not able to succeed academically or perhaps socially without such technology. Thus, financial support and COVID-19 benefits should be prioritized to low-income families with adolescents to improve family relationships, particularly between parents and their children. That is, during COVID-19, improving economic status among low-income families might be beneficial to increase family satisfaction, leading to increase self-efficacy among low-income students.

This study's findings also indicated that parents' educational attainment in low-income families moderated the association between family satisfaction and self-efficacy among adolescents. Educational attainment among parents is one important way to help their children develop high self-efficacy (11, 12, 36). This study showed that 39.6% of parents in low-income households received higher education. Thus, in more than half of low-income families, both parents had not received higher education. As the current study demonstrated that low-income students whose both parents received higher education consistently reported higher levels of self-efficacy, the group of low-income students with at least one parent who did not receive higher education had lower self-efficacy. This phenomenon may be worsened during COVID-19 because low-income students have had fewer chances to access additional resources such as after school programs or in-person tutoring due to tight COVID-19 restrictions and have suffered from negative academic, nutrition and mental health impacts as a result (37).

Moreover, as students who are satisfied with their family relationships tend to have higher self-efficacy, greater family satisfaction may buffer against low self-efficacy among low-income students whose parents did not receive higher education. This study confirmed that the effect of family satisfaction on self-efficacy was greater for the group of low-income students without a parent who received higher education vs. those with at least one parent who received higher education. Therefore, social welfare programs targeting family relationships in low-income households should be especially targeted toward low-income households without a parent who received higher education. Further, low-income households have encountered more financial strains during COVID-19, perhaps leading parents to have to work more h and thus not have as much time to communicate with their children, resulting in lower levels of family satisfaction. Thus, financial support such as COVID-19 wage subsidies, job stability fund programs, and employee retention subsidies should be expanded to financially help low-income parents. in order to maximize the effect of family satisfaction on self-efficacy among low-income students, and these benefits should be focused on low-income families in which neither parent received a higher education. On the other hand, this study demonstrated that low-income students with two parents who received a higher education were more likely to have higher self-efficacy. Thus, life-long education should be provided to parents in low-income families who did not gain higher education as their educational attainment influences the self-efficacy of their adolescent children. Further, to address inequalities in children's psychological development, more opportunities for higher education should be provided to low-income students to help them increase their income and economic status into adulthood.

## Conclusion and limitations

Although it is important to help adolescents to develop self-efficacy, little has been studied about self-efficacy among low-income students since COVID-19. This study contributes to understanding how to increase self-efficacy among low-income adolescents since COVID-19 by considering their parents' educational attainment. However, this study has a few limitations that must be considered. First, the results may be applied to other countries, especially Asian countries which have similar attributes of economics, politics, and culture. However, we suggest that before findings are examined in the context of other cultures, that such cultural differences should be considered. Second, even though this study used a nationwide sample, the participants were limited to students who were registered in a mentorship program. This study controlled for level of satisfaction with the mentorship program, but the sample might not be a representative of or generalizable to all low-income students, as low-income students not enrolled in the mentorship program were not surveyed. Third, for educational attainment, we classified it into two groups: both parents whose educational level was greater than high school and those who were not. This classification might limit effect of educational attainment among some groups in which one of parents received higher education.

## Data availability statement

The raw data supporting the conclusions of this article will be made available by the authors, without undue reservation.

## Ethics statement

The Institutional Review Board of Inha University approved this study (#210216-2A). Written informed consent to participate in this study was provided by the participants' legal guardian/next of kin.

## Author contributions

All authors listed have made a substantial, direct, and intellectual contribution to the work and approved it for publication.

## Conflict of interest

The authors declare that the research was conducted in the absence of any commercial or financial relationships that could be construed as a potential conflict of interest.

## Publisher's note

All claims expressed in this article are solely those of the authors and do not necessarily represent those of their affiliated organizations, or those of the publisher, the editors and the reviewers. Any product that may be evaluated in this article, or claim that may be made by its manufacturer, is not guaranteed or endorsed by the publisher.
